# Maternal Obesity Induces DNA Methylation Changes in the Kidneys and Blood of Rodent Offspring at Adulthood

**DOI:** 10.3390/nu18193212

**Published:** 2026-09-29

**Authors:** Benjamin P. Larkin, Long T. Nguyen, Naveen K. Parthiban, Hui Chen, Alen Faiz, Kushalee Jayawickreme, Sarah Glastras, Carol A. Pollock, Sonia Saad

**Affiliations:** 1 Renal Research Laboratory, Level 9, Kolling Institute of Medical Research, Royal North Shore Hospital, University of Sydney, St. Leonards, Sydney, NSW 2065, Australia; benjamin.larkin@health.nsw.gov.au (B.P.L.); long.t.nguyen@sydney.edu.au (L.T.N.); kushalee.jayawickreme@health.nsw.gov.au (K.J.);; 2 School of Life Sciences, Faculty of Science, University of Technology Sydney, Ultimo, Sydney, NSW 2007, Australia; 3 Department of Diabetes, Endocrinology and Metabolism, Royal North Shore Hospital, St. Leonards, Sydney, NSW 2065, Australia

**Keywords:** maternal obesity, chronic kidney disease, fetal programming, DNA methylation, epigenetics

## Abstract

Aim: To identify differentially methylated genes in offspring of obese versus lean mothers using a mouse model of maternal obesity. Background: Adverse conditions during fetal development predispose offspring to adult diseases, supporting the theory of fetal programming. DNA methylation is suggested as a mechanism by which maternal obesity induces fetal programming. As maternal obesity predisposes offspring to chronic kidney disease (CKD), we aimed to investigate the role of DNA methylation in maternal obesity related to CKD in the offspring. Methods: Maternal obesity was modelled by feeding female C57BL/6 mice a high-fat diet before breeding, and during gestation and lactation. Control dams received chow. Male offspring were weaned to chow on postnatal day 20 and were sacrificed at 32 weeks of age (adulthood). Another cohort was sacrificed at birth. Reduced representation bisulfite sequencing was used to identify differentially methylated genes (DMGs) in the offspring kidney and blood at adulthood and at birth. Real-time PCR was used to determine gene expression. Results: Adult offspring of obese dams showed significant DMGs in both kidney and blood at adulthood in association with increased levels of kidney injury. Among these, 10 genes were differentially methylated in the kidney, out of which, four genes were also differentially methylated in the blood. An additional 10 genes were differentially methylated in the blood only. Renal mRNA expression of four genes (*Rcsd1*, *Cdk8*, *Taco1* and *Axin2*) was significantly upregulated, confirming transcriptional alteration of these genes in the offspring due to epigenetic regulation. Pathway analysis revealed enrichment of genes involved in the Wnt canonical signalling pathway. In comparison, no significant DMGs were detected at day 1. Conclusions: Maternal obesity induced differential gene methylation in the kidneys and blood of adult offspring. Several of these genes have shown high relevance to kidney fibrosis, diabetes or obesity, supporting the role of epigenetic regulation in fetal programming to CKD by maternal obesity.

## 1. Introduction

It has recently been established that maternal obesity is also a significant risk factor for the development of CKD in offspring in later life [1]. This is in keeping with the notion of fetal programming, which suggests that adverse exposures occurring in utero predispose the fetus to chronic diseases in adulthood [2,3]. Using rodent models of maternal obesity, we have previously demonstrated that offspring exposed to maternal obesity during gestation develop features of CKD including albuminuria, glomerulosclerosis and renal fibrosis at adulthood, as well as increased renal oxidative stress and inflammation [4,5].

Epigenetic modifications are heritable changes which affect gene expression without altering the underlying DNA sequence, and include DNA methylation, histone modification and RNA interference [6,7]. Of these, DNA methylation is the most extensively studied epigenetic signature [8]. DNA methylation may occur in response to environmental stressors such as diet, physical activity, drugs, toxins and infections [8,9,10]. However, as the majority of DNA methylation occurs in mammals during the in utero period, coinciding with critical periods of fetal development, DNA methylation has been proposed as a mechanism through which fetal programming of adult chronic disease occurs [11,12,13,14].

Both animal and human studies have identified differences in DNA methylation levels in the offspring of obese versus lean mothers. In a mouse maternal obesity model, differentially methylated genes have been identified in the livers of week-8 offspring born to obese versus lean mothers [15]. Livers of week-8 offspring exposed to maternal obesity had hypermethylation of the insulin receptor substrate 2 (*Irs2*) gene, and hypomethylation of the mitogen-activated protein kinase 4 (*Map2k4*) gene, which are suggested to predispose offspring to diabetes later in life [15]. In a human study, exposure to maternal obesity increased methylation of the leptin promoter gene on the fetal side of the placenta [16]. Given that leptin helps establish nutritive, secretory and anchoring functions of the placenta, this finding suggests that maternal obesity alters placental functions through epigenetic mechanisms [16]. In a human epigenome-wide analysis, multiple differentially methylated loci were identified in the cord blood leucocytes of offspring of obese versus non-obese mothers [17]. In a large meta-analysis of 9340 mother–newborn pairs across 19 cohorts, robust associations between maternal obesity and DNA methylation were identified at 86 CpG sites in neonatal whole blood. Four independent cohorts subsequently underwent DNA methylation analysis of whole blood at adolescence, and several of the differentially methylated loci detected in newborns were reproduced in adolescents [18].

To our knowledge, the effect of maternal obesity on DNA methylation in the kidneys of offspring has not been reported until our recent studies [19,20], which showed renal global DNA methylation was upregulated in the offspring at week 32 due to maternal obesity. Meanwhile, DNA methylation has been associated with several aspects relevant to CKD. In human and animal studies, differentially methylated genes within the kidney and blood have been identified between CKD cases and controls [21,22,23,24,25,26,27]. Additionally, differential DNA methylation patterns have been observed in whole blood between patients with rapidly progressive CKD and those with stable CKD [28]. Further, in patients with CKD, associations have been drawn between uraemia, infection, inflammation and patterns of DNA methylation in peripheral blood leucocytes [29,30,31].

Differences in DNA methylation may explain mechanisms by which maternal obesity programmes CKD. Therefore, we hypothesized that DNA methylation occurring in the kidneys of the offspring of obese mothers may be linked to CKD in adulthood. Using a mouse model of maternal obesity, we aimed to identify differentially methylated genes between adult offspring exposed to maternal obesity, and control offspring as well as DNA methylation in the offspring at birth. A post hoc analysis of differentially methylated genes was conducted, specifically identifying gene loci with relevance to CKD and metabolic conditions, validating changes in gene expression secondary to the differentially expressed methylation patterns between the two groups.

## 2. Materials and Methods

### 2.1. Animal Experiments

The study was approved by the Animal Ethics Committee of the Northern Sydney Local Health District (Resp/17/29) and complied with the Australian code for the care and use of animals for scientific purposes, and took place at Kearns Animal Facility. After 1 week of acclimatization, 8-week-old female mice were randomized to either a high-fat diet (HFD, 20 kJ/g, 43% of total energy from fat [Specialty Feeds, Glen Forrest, WA, Australia]) to induce maternal obesity or chow as previously described [19,20,32]. Breeding began at 14 weeks of age and the dams were maintained on the same diet throughout pregnancy and during lactation. On postnatal day 1, offspring were either culled to collect blood and kidney tissue, or their litter size was reduced to four pups for breastfeeding to avoid differences in milk competition. Male offspring were weaned to a chow diet on postnatal day 20. There were two offspring groups: control offspring (CC) and offspring exposed to maternal obesity (HC). At 32 weeks, animals were euthanized under anesthesia (isoflurane, 2%). For assessment, each pup was selected from a different litter from a different dam to ensure each biological replicate is independent from each other. Hence, the number dams/litters used in this study are equal to the sample size/experiment. Four animals/group were used for day 1 and seven to eight animals per group were used for week 32, among which three animals were used for DNA methylation analysis. Blood was collected via cardiac puncture. Blood was collected in EDTA-tube and kidneys were snapped frozen for DNA extraction. All animal procedures were performed in accordance with the Australian Code for the Care and Use of Animals for Scientific Purposes.

### 2.2. Urine Measures

A mouse albumin ELISA kit (Crystal Chem, Itasca, IL, USA) was used to quantify urinary albumin concentration, and creatinine concentration was determined using a colorimetric assay kit (Cayman Chemical, Ann Arbor, MI, USA). The urinary albumin:creatinine ratio was then calculated (*n* = 7).

### 2.3. Renal Structural Changes

Formalin-fixed kidneys were sectioned (2 μm thickness). Periodic Acid Schiff (PAS) staining and picrosirius red (PSR) were performed (6 samples per group). Light microscopy (Leica, Wetzlar, Germany) was used to assess for glomerulosclerosis and tubulointerstitial injury, scored by two independent, blinded investigators. Glomeruli were graded on a scale of 0 to 4 as follows: (0: normal; 1: involvement of <25% of the glomerulus; 2: involvement of 25–50% of the glomerulus; 3: involvement of 50–75% of the glomerulus; 4: totally sclerosed) [33]. The glomerulosclerosis index was calculated by averaging the scores of 20 glomeruli per slide. Tubulointerstitial injury was assessed on PSR staining as a composite score of tubulointerstitial fibrosis, tubular vacuolation, tubular dilatation and casts, as previously described [32].

### 2.4. DNA Methylation Study

Genomic DNA was extracted from kidney tissue and whole blood using the DNeasy Blood and Tissue Kit (Qiagen Pty Ltd., Redwood City, CA, USA). Whole kidney was used at day 1 and a section of the kidney cortex was used at week 32. An amount of 100 ng of DNA was used as the input. RRBS was performed by the Australian Genome Research Facility (AGRF, Melbourne, Australia) using Illumina NovaSeq sequencing platform. Briefly, the RRBS library was produced using the NuGEN Ovation^®^ RRBS Methyl-Seq system (30 million reads, 100 bp single end run) and assessed using FastQC v.0.11.8 [34] and adapter trimming using Trim Galore! [35]. Bisulfite-converted RRBS libraries yielded an average of 32.5 million reads per sample, with consistent GC content (29–30%) and read length (~78–80 bp) across all libraries, confirming successful size selection for CpG-enriched fragments. Reads were aligned to the GRCm38 mouse genome using Bismark/Bowtie2, achieving a mapping efficiency of ~80%). Non-conversion rate, estimated from unmethylated lambda DNA spike-in, was uniformly low across all samples, confirming complete and efficient bisulfite conversion. Library sizes were limited to loci with coverages ≥ 8, before calculating beta and M values [36]. To avoid zero and infinite values in M value computation, the offset value (α) was set at 0.1. Reads were aligned to the mouse reference genome using Bismark [37] with Bowtie 2 [38], and differential methylation analysis was performed using the edgeR-based pipeline for RRBS [39,40].

### 2.5. Quantitative Real-Time PCR (RT-PCR)

RNA was extracted from kidney tissue (*n* = 6–8) using the RNeasy Plus Mini Kit (Qiagen, Redwood City, CA, USA). cDNA was generated using the iScript cDNA Synthesis Kit (Biorad, CA, USA). Mastermixes incorporating Quantinova SYBR Green (Qiagen) and primers listed in Supplementary Table S1 were used to perform RT-PCR with the QuantStudio 12K Flex-Real Time PCR System (Thermo Fisher Scientific, Waltham, MA, USA). Primers were designed for the target genes and commercially synthesized by Sigma-Aldrich (Merck, Macquarie Park, NSW, Australia). Primer specificity was initially assessed by amplification of the target sequence, followed by melt-curve analysis. A single, distinct melting peak was observed for each primer pair, consistent with the amplification of a single specific PCR product and the absence of detectable non-specific amplification or primer-dimer formation. Primer pairs demonstrating a single specific amplification product were subsequently used for quantitative real-time PCR (qRT-PCR) analysis as we have previously described [19,20,32] Results are expressed as fold change after normalization to 18S.

### 2.6. Statistical Analysis

All results, with the exception of the methylation analysis, are represented as mean ± SEM. Statistical analysis was performed using unpaired *t*-tests (GraphPad Prism v8.3.1, San Diego, CA, USA). Significant results had *p* values of <0.05. For DNA methylation data, post-sequence bioinformatic analysis was conducted in R (version 4.3.0) using RStudio with the tidyverse suite of packages (version 2.0.0) [41]. Differential methylation was quantified at the CpG locus level via Benjamini–Hochberg false discovery rate (FDR) correction. Loci with FDR < 0.05 were deemed statistically significant. Several critical CpG loci per gene were further collapsed to gene-level summaries using the most relevant locus per gene. Volcano plots were calculated using ggplot2 (version 4.0.3) and ggrepel (version 0.9.8). Gene ontology (GO) biological process and KEGG pathway enrichment analyses were performed using clusterProfiler ORA (over-representation analysis) (version 4.18.4) (Yu et al., 2012), with org.Mm.eg.db (version 3.22.0) as an annotation database (Carlson, 2019) [42,43].

## 3. Results

### 3.1. Maternal HFD Induced Kidney Damage in the Offspring

Male offspring born HFD-fed dams showed no change in body weight at 32 weeks. However, kidney weight to body weight ratio was significantly increased with age (*p* < 0.01, Figure 1C). UACR was also significantly increased in these offspring compared to those born to chow-fed dams (*p* < 0.05, Figure 1D). This was associated with an increase in glomerular sclerosis (*p* < 0.05, Figure 1E) and tubulointerstitial injury composite indices (*p* < 0.05, Figure 1F).

### 3.2. Genes Differentially Methylated in the Kidney at Week 32

In the kidney, there were 10 genes in total with significant differences in methylation status between offspring of obese mothers and controls (Figure 2A, Supplementary Table S2). Of these, three genes were significantly hypermethylated, including the RIKEN cDNA 2810004N23 gene (*2810004N23Rik*), ring finger protein 150 (*Rnf150*) and the cell adhesion-associated, oncogene-regulated gene (*Cdon*). Seven genes were significantly hypomethylated, including, RSCD domain-containing 1 (*Rcsd1*), cyclin-dependent kinase 8 (*Cdk8*), peroxisomal biogenesis factor 2 (*Pex2*), Translational Activator of Cytochrome C Oxidase I (*Taco1*), *Axin2*, the RIKEN cDNA 1700030C10 gene (*1700030C10Rik*) and predicted gene 20110 (*Gm20110*). Among differentially methylated genes in the offspring kidney due to maternal obesity, *Axin2* had the most differentially methylated loci (nine loci), followed by *Cdk8* and *Pex2* (eight loci each) (Supplementary Table S2). Meanwhile, five genes including *Rnf150*, *2810004N23Rik*, *Cdon*, *Taco1* and *Gm20110* had differential methylations at a single locus.

### 3.3. Genes Differentially Methylated in the Blood at Week 32

In the blood, there were also 14 genes with significant differences in methylation status between offspring of obese mothers and controls (Figure 2B, Supplementary Table S3). Of these, eight were significantly hypermethylated in offspring of obese mothers compared to control offspring: *2810004N23Rik*, *Arhgef26*, *Ets1*, *Cdk8*, *Gm28590*, *Cylc1*, *H2-Q6* and *Tmtc2*. Six genes were significantly hypomethylated: *Rcsd1*, *Pex2*, *Pcdh7*, *SOCS3*, *B230209E15Rik* and *A630010A05Rik*. Particularly, *SOCS3*, *Cdk8*, *Rcsd1* and *Arhgef26* were differentially methylated at multiple loci (six, six, five and two, respectively), while all other genes were differentially methylated at the single locus (Supplementary Table S3). Between kidney and blood, four genes including *Rcsd1*, *Cdk8*, *Pex2* and *2810004N23Rik* were commonly regulated, among these *Rcsd1*, *Pex2* and *2810004N23Rik* showed consistent directions of methylation.

### 3.4. mRNA Expression with Differential Methylation in the Kidney Only

*Rnf150* mRNA expression in the kidney was similar between the offspring of obese mothers and controls (Figure 3A). Offspring of obese mothers had significantly increased renal mRNA expression of *Taco1* and *Axin2* compared to offspring of chow-fed mothers (*p* < 0.05 for both, Figure 3B,C). Renal mRNA expression was similar between groups for *1700030C10Rik*, *Gm20110* and *Cdon* (Figure 3D–F). Supplementary Table S4 correlates gene methylation findings with renal mRNA expression in the kidney.

### 3.5. mRNA Expression of Common Differentially Methylated Genes in Kidney and Blood

Kidney mRNA expression of *Rcsd1* and *Cdk8* was significantly increased in the offspring of obese mothers versus controls (*p* < 0.05 for both, Figure 4). There was a trend towards increased mRNA expression of *Pex2* in the offspring of obese mothers compared to controls; however, this did not reach statistical significance (*p* = 0.12, Figure 4). Renal *2810004N23Rik* mRNA expression was similar between groups. Supplementary Table S5 correlates gene methylation findings in both the kidney and blood with renal mRNA expression.

### 3.6. Pathway Analysis at Week 32

Pathway analysis of methylated genes in the offspring kidney at week 32 revealed significant enrichment of pathways related to Wnt signalling regulation at the gene ontology level (Figure 5). In the blood, pathways related to inflammatory responses and lipid metabolism were significantly enriched. Common pathways indicate peroxisome regulation in both the blood and kidney. Consistently, KEGG pathway analysis also suggests modest enrichment of Wnt signalling and peroxisome regulation in the offspring kidney. Meanwhile, pathways related to Type 1 and type 2 diabetes were significant in the blood.

### 3.7. DNA Methylation at Postnatal Day 1 in Comparison to Week 32

There were no significant differentially methylated genes in both the kidney and blood of the offspring at postnatal day 1 (FDR > 0.05). However, when looking at the methylation levels of the aforementioned significant genes at week 32 in offspring kidney at day 1, the directions of methylation were consistent for six genes including Axin2, Cdk8, Pex2, *1700030C10Rik*, *Rcsd1*, and *Gm20110* (hypomethylation) (Figure 6). Similarly, *Cdk8*, *Arhgef26***,** *Gm28590*, *Tmtc2 2810004N23Rik*, *H2-Q6*, *Cylc1* and *Ets1* showed the same tendency to hypermethylation in the blood at day 1 as they did at week 32. In addition, *SOCS3* showed tendency to hypomethylation in the offspring blood at day 1 and week 32.

### 3.8. Pathway Analysis at Day 1

Pathway analysis of top 100 genes in the offspring kidney at day 1 indicates enrichment of kidney-development-related pathways including nephron tubule formation, mesonephric tubule/epithelium development, ureteric bud development, kidney/renal system development, and kidney epithelium development (Figure 7). This signature is distinct from regulated pathways in the blood, which seems to highlight those related to changes such as aortic valve morphogenesis, heart valve morphogenesis, dendrite morphogenesis, VEGF signalling, and lipid metabolism. In KEGG, the kidney also showed enrichment of Wnt signalling, which is consistent with changes at week 32. To a lesser extent, Foxo signalling was also regulated in the kidney. In comparison, amino acid and lipid metabolism pathways (tryptophan, arginine/proline, histidine, fatty acid degradation, cholesterol metabolism) dominate regulated pathways in the blood.

## 4. Discussion

In this study, we demonstrated that maternal obesity induced DNA methylation changes in the kidneys and blood of mouse offspring in adulthood, which were associated with increased levels of kidney injury. Pathway analysis revealed significant enrichment of inflammatory and metabolic pathways in the blood, and Wnt signalling in the offspring kidney at week 32. In comparison, the DNA methylation profiles of offspring at day 1 indicated enrichment of the kidney and cardiovascular systems, as well as lipid metabolism. These findings support the hypothesis that DNA methylation plays a role in the mechanism of developmental programming of CKD in offspring at adulthood.

The DNA methylation profile is usually attributed to the interaction between genomes and environmental factors [44]. In this case, neonatal exposure to maternal high-fat diet is the modifying factor. As the offspring were exposed to maternal HFD during gestation and lactation and then weaned to chow, it is logical to assume that any modification that occurred due to maternal obesity needed to persist until adulthood to be capable of mediating the development of CKD. Interestingly, in our study, there were 10 and 14 significant DMGs in the kidney and blood at week 32 but no significant DMGs at postnatal day 1. These results reflect the fact that DNA methylation is a dynamic process that is unlikely to remain unchanged from puberty to adulthood. The indirect mechanism and the small sample size may also underline the lack of significant changes at day 1. That said, as a great proportion of DMGs at week 32 showed the same direction of regulation at day 1 and, additionally, by using the top 100 DMGs as per nominal *p*-values for day 1 pathway analysis, we identified enrichment of several pathways that were also significant at week 32. This finding, although exploratory, suggests that early changes in DNA methylation may occur at pathway levels that are persistent or recurrent in adulthood. Further studies with increased sample size are warranted to confirm this hypothesis.

Among different pathways that were regulated in the kidney, the Wnt signalling-relevant pathways stood out as they appeared significant at both day 1 and week 32, and in both GO and KEGG analysis. This supports the hypothesis that certain epigenetic traits related to the fetal programming effects of maternal obesity were maintained in the offspring kidney until adulthood, contributing to the development of CKD. Indeed, genes of the Wnt signalling pathway have also been found to be differentially methylated in HFD-fed mice which develop progressive CKD [44]. As a key negative feedback regulator in this pathway, *Axin2* showed downregulated DNA methylation and upregulated mRNA expression in the offspring kidney due to maternal obesity. Another DMG involved in the canonical Wnt signalling pathway is *Cdon*, a cell adhesion protein known for its suppression effects on this signalling cascade, the role of which has not been reported in the kidneys. In this study, *Cdon* was hypermethylated in association with a trend to increase in mRNA expression. When the kidney is injured, the Wnt pathway is usually activated to promote cell growth for tissue regeneration. The increased mRNA expression of *Axin2* and *Cdon* was likely to inhibit this mechanism, leading to maladaptive repair. In line with our findings, other studies have shown that kidney expression of *Axin2* was significantly increased in other CKD models including the unilateral ureteral obstruction (UUO) model [45], and diabetic glomerulosclerosis [46].

Differential gene methylation of *Cdk8* was found in the kidney and the blood, even though it was hypomethylated in the kidney, and hypermethylated in the blood. The discrepancy in the pattern of gene methylation between the kidney and blood is not surprising, given that CDKs have roles in cell differentiation and proliferation [47], which do not occur in mature circulating blood cells. The hypomethylation of *Cdk8* in the kidney was associated with increased mRNA expression in the kidneys of offspring of obese mothers compared to controls. *Cdk8* is known to phosphorylate the Notch intracellular domain, which activates the Notch signalling pathway [48]. We have previously demonstrated that Notch signalling is involved in renal fibrosis [49]. Similar to Wnt signalling pathway, the Notch signalling pathway also plays critical roles during kidney development, which is generally silenced once maturation is complete [50]. In rodent models, sustained activation of Notch signalling after kidney development has been associated with the development of tubulointerstitial fibrosis, glomerulosclerosis and albuminuria [50,51]. Decreased methylation in the kidney supports its role in kidney fibrosis via Notch pathway activation. *Cdk8* also phosphorylates and therefore inhibits sterol regulatory element-binding proteins (SREBPs), which are transcription factors regulating the expression of genes involved in lipid synthesis [52], and have been implicated in the pathogenesis of obesity, T2D, atherosclerosis, CKD and non-alcoholic fatty liver disease [53].

Apart from the genes discussed above, *Taco1*, *Rscd1* and *Pex2* were the other genes with DNA hypomethylation and increased mRNA expression in the offspring kidney due to maternal obesity. *Taco1* is involved in mitochondrial respiration, and encodes a protein which is required for the translation and synthesis of subunit I of cytochrome c oxidase [54]. *Rcsd1* plays a role in regulating actin filament binding, a process crucial for cells to migrate, feed and divide [55], while *Pex2* encodes a peroxisomal membrane protein, involved in the biosynthesis of peroxisomes [56], which are membrane-bound organelles crucial to the oxidative metabolism of lipids [57]. *Rscd1* is usually found in immune cells, hence its increased expression in the kidney may suggest immune cell infiltration in kidney tissue. That said, our previous study on the same model did not determine significant changes in the mRNA expression of inflammatory markers MCP-1 and CD68 in the kidney of offspring born to HFD-fed dams [19,20]. The study, however, did demonstrate significant increases in the expression of oxidative stress markers, which is consistent with the upregulation of *Pex2*. The fact that *Rscd1* and *Pex2* showed the same direction of methylation and mRNA expression in both the kidney and blood supports the same mechanism of regulation, albeit the precise mechanism of their involvement in the developmental origin of kidney disease remains to be elucidated.

Among CpG loci that show differential methylation at week 32, several belong to non-coding regions including *1700030C10Rik*, *Gm20110*, *B230209E15Rik*, *A630010A05Rik* and *Gm28590*. The fact that we detected no changes in the renal mRNA expression of 2810004N23Rik,1700030C10Rik and Gm20110 due to maternal obesity suggest that these methylation sites do not regulate transcriptional expression. Similarly, several of the genes found to be significantly differentially methylated between the offspring of obese and lean mothers have functions which are unrelated to CKD, diabetes or obesity. The significance of these genes in maternal obesity-related CKD is unclear and also warrants further investigations.

There are a number of limitations of this study which warrant acknowledgement. This study utilized a relatively small sample size. Despite this, we demonstrated highly significant methylation differences in several genes between the offspring of obese versus lean mothers. To validate findings, future studies could be performed using increased sample sizes. Further, although we have previously demonstrated that offspring of obese mothers are predisposed to CKD [4,32], in the current study, the methylation differences arising due to maternal obesity suggest that maternal obesity programmes CKD through these mechanisms. Future studies using knockdown models could help identify the roles of these candidate genes in the fetal programming of CKD by maternal obesity.

It is important to acknowledge that the tissues used in our methylation analysis (kidney cortex and whole blood) are composed of multiple cell types. It has been reported that samples derived from heterogeneous mixtures of cells have the potential to confound DNA methylation results depending on the relative proportions of cell-types present [58]. Deconvolution studies can be performed to adjust for potential confounding by cell-type, and, although beyond the scope of the current study, these could be a focus of future investigations.

## 5. Conclusions

Our analysis of mouse kidney and blood samples revealed highly significant gene methylation differences at adulthood between offspring exposed to maternal obesity and controls, which are related to the Wnt signalling pathway. Maternal obesity-induced DNA methylation changes in offspring have not previously been reported in kidney tissue. Upon review of the literature, several of the differentially methylated genes were noted to have high relevance to CKD, fibrosis, diabetes, obesity and lipid metabolism, supporting the hypothesis that epigenetic regulation of these genes may be involved in the fetal programming of CKD by maternal obesity. Further studies on a larger cohort and in human tissues are required to confirm this mechanism.

## Figures and Tables

**Figure 1:**
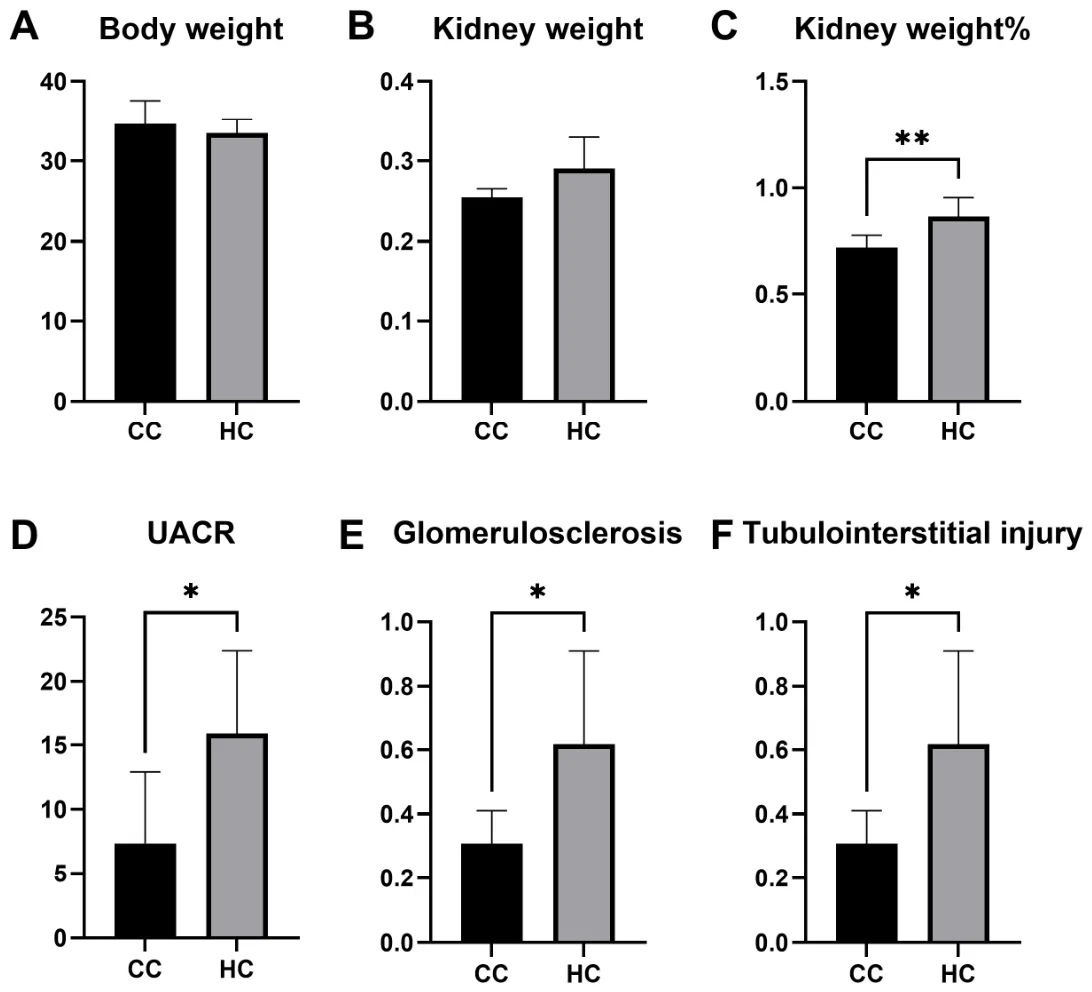
Offspring body weight, kidney weight and pathology scoring. (**A**) Body weight, (**B**,**C**) kidney weight, (**D**) urinary albumin creatinine ratio, (**E**,**F**) kidney histological assessment. *n* = 6–8. * *p* < 0.05, ** *p* < 0.01.

**Figure 2:**
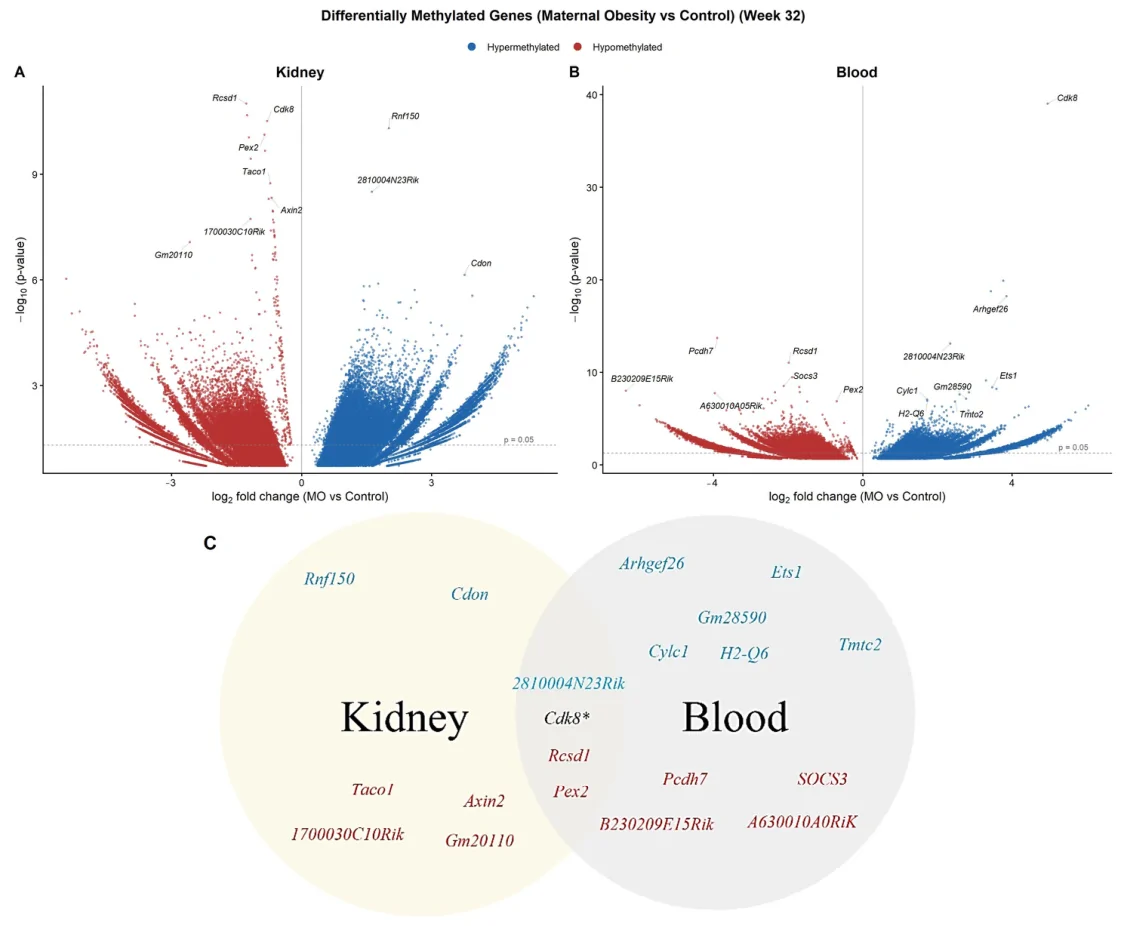
Differential DNA methylation in offspring’s kidney and blood at week 32. Volcano plots of hypomethylated (red) and hypermethylated (blue) in (**A**) kidney, (**B**) blood. (**C**) Venn diagram showing common genes between blood and kidney. * Cdk8 was hypermethylated and hypomethylated in blood and kidney, respectively. FDR < 0.05 was deemed significant. *n* = 3/group.

**Figure 3:**
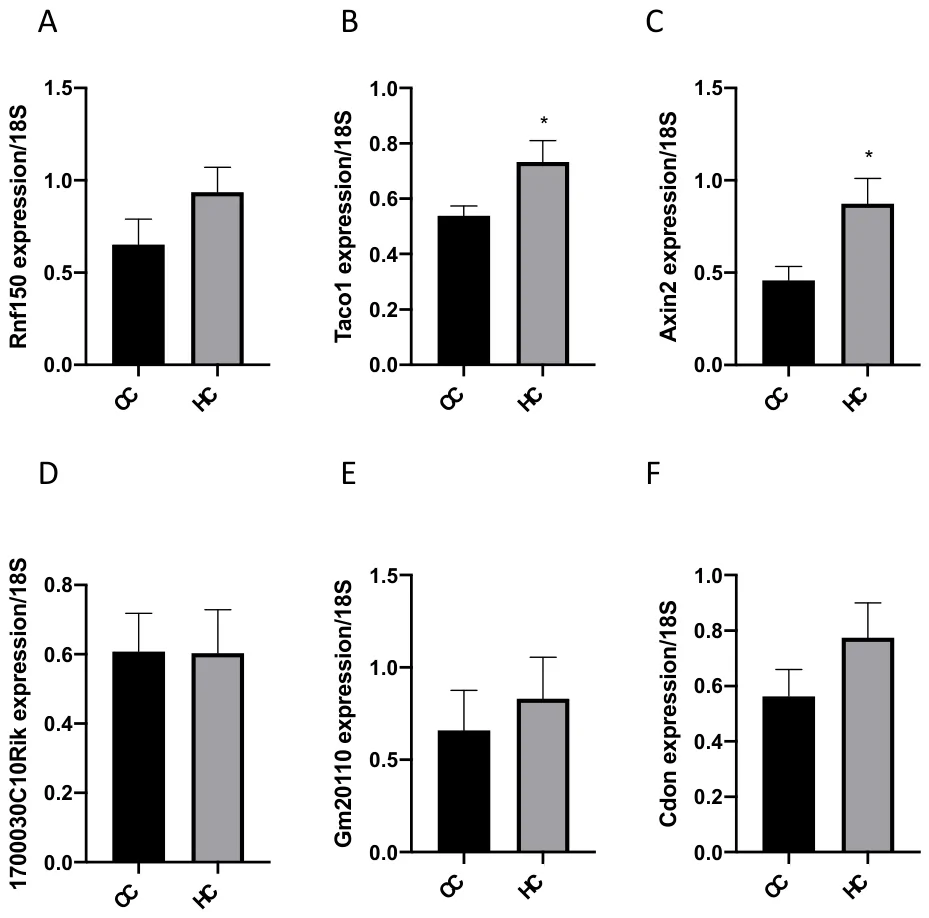
Kidney mRNA expression of genes identified to be differentially methylated due to maternal obesity in the kidney only. (**A**) mRNA expression of *Rnf150*, (**B**) mRNA expression of *Taco1*, (**C**) mRNA expression of *Axin2*, (**D**) mRNA expression of *1700030C10Rik*, (**E**) mRNA expression of *Gm20110*, (**F**) mRNA expression of *Cdon*. Results expressed as mean ± SEM, *n* = 6–8, * *p* < 0.05. CC: control offspring; HC: offspring exposed to maternal obesity.

**Figure 4:**
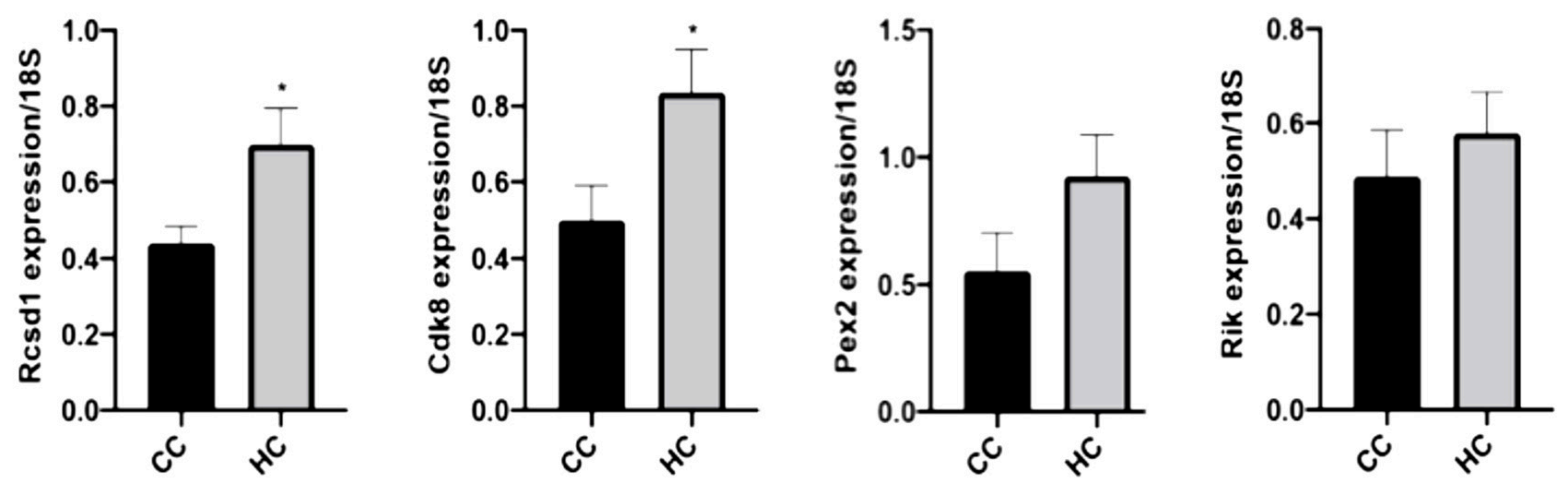
Kidney mRNA expression of genes identified to be differentially methylated due to maternal obesity in the kidney and blood. Results expressed as mean ± SEM, *n* = 6–8, * *p* < 0.05. CC: control offspring; HC: offspring exposed to maternal obesity.

**Figure 5:**
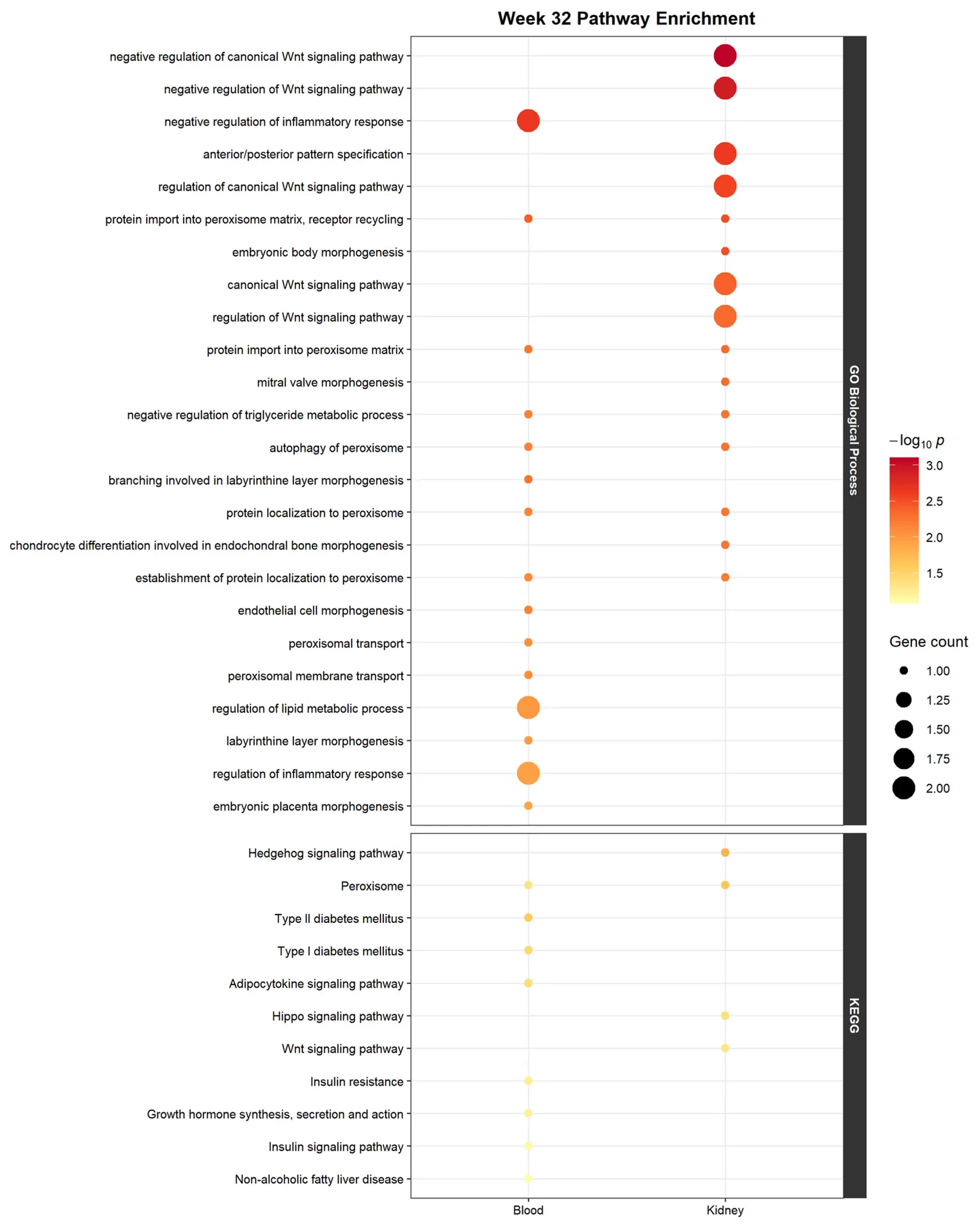
The pathway enrichment analysis of the most significantly differentially methylated genes at week 32. A Gene Ontology Biological Process and KEGG pathway over-representation analysis was conducted on significant differentially methylated genes, ranked by nominal *p*-value, in the blood and kidney of week 32 adult offspring exposed to maternal obesity, in comparison to control offspring. The size of each dot indicates the number of genes contributing to a given pathway, while the dot colour signifies the intensity of enrichment (expressed as −log_10_ *p*-value). Key enriched pathways identified include negative regulation of Wnt signalling, peroxisome function, inflammatory response, and cellular senescence, primarily influenced by genes such as Axin2, Cdon, Pex2, and Socs3. *p*-value < 0.05 is considered significant.

**Figure 6:**
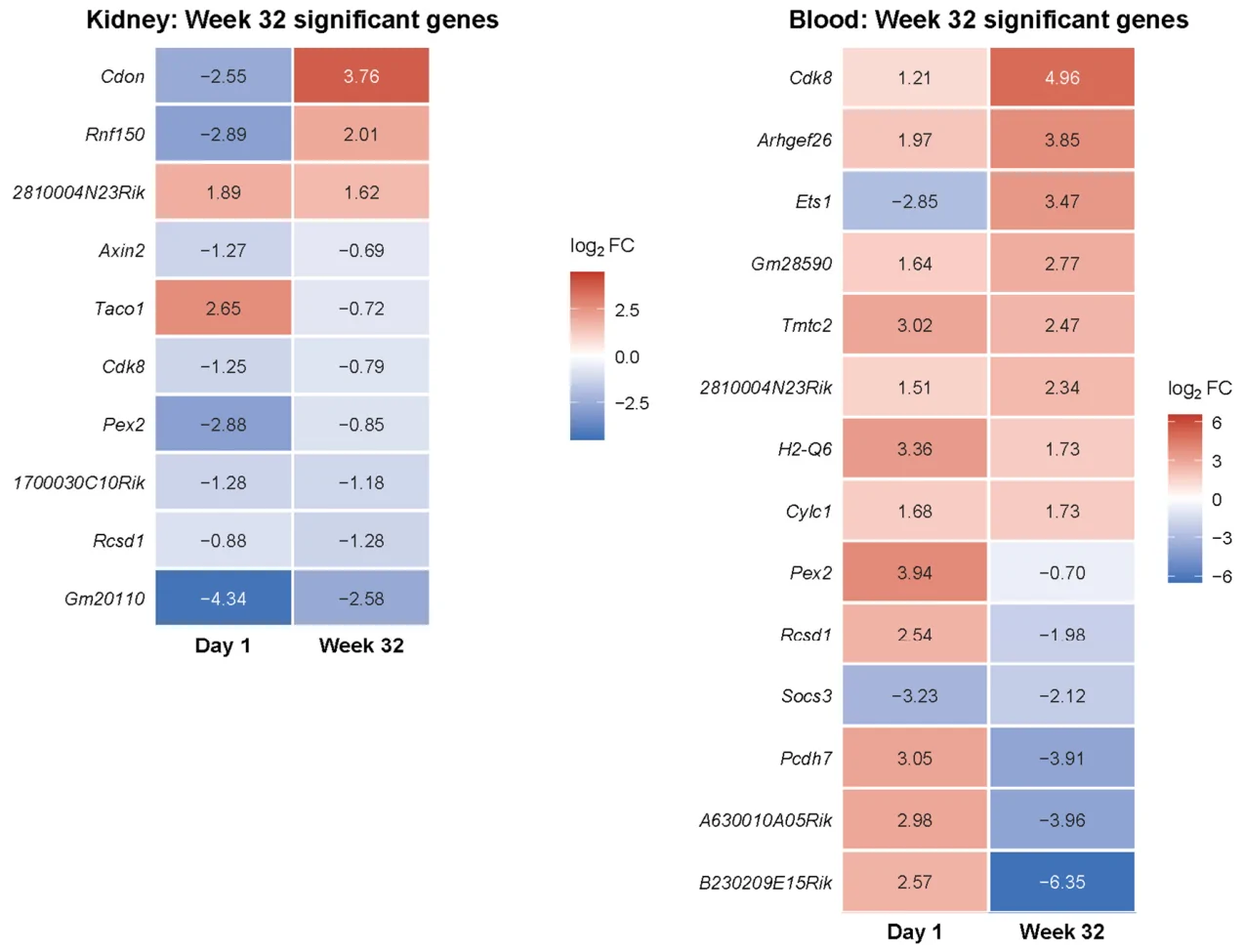
Comparison of DNA methylation changes at day 1 to week 32 in kidney and blood. Log_2_FC > 0 means hypermethylation; Log_2_FC < 0 means hypomethylation.

**Figure 7:**
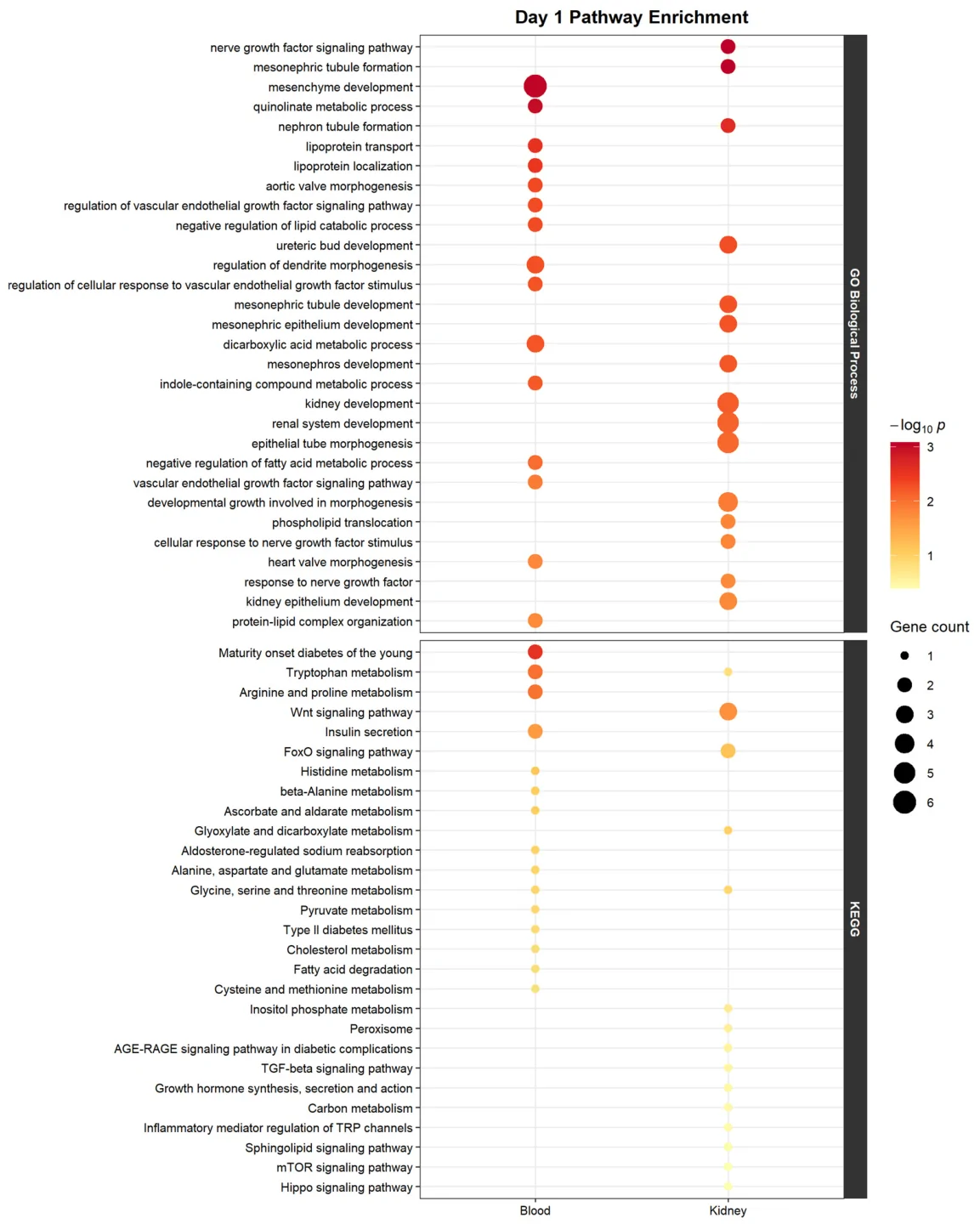
Pathway enrichment analysis of top-ranked differentially methylated genes at postnatal day 1. Gene Ontology Biological Process and KEGG pathway over-representation analysis was performed on the top 100 differentially methylated genes, ranked by nominal *p*-value, in the blood and kidney of postnatal-day-1 offspring exposed to maternal obesity versus control offspring. Dot size represents the number of genes contributing to each pathway, while dot colour indicates enrichment intensity (−log_10_ *p*-value). The most enriched pathways included Wnt signalling, actin cytoskeletal regulation, and ribosome biogenesis, primarily driven by genes such as Acta2, Rcsd1, Nop2, and Cat. *p*-value < 0.05 is considered significant.

## Data Availability

Data are available upon request.

## Supplementary Materials

The following supporting information can be downloaded at: https://www.mdpi.com/article/10.3390/nu18193212/s1, Table S1: Mouse-specific primers used in quantitative real-time PCR; Table S2: Genes with differential methylation identified in the kidney; Table S3: Genes with differential methylation identified in the blood; Table S4: Summary of methylation findings and kidney mRNA expression of genes identified to be significantly differentially methylated due to maternal obesity only in the kidney; Table S5: Summary of methylation findings and kidney mRNA expression of genes identified to be significantly differentially methylated in both the kidney and blood due to maternal obesity.

## Abbreviations

BH-FDR, Benjamini–Hochberg false discovery rate; BMI, body mass index; CB, control blood; CC, control offspring (chow-fed dam); CKD, chronic kidney disease; CpG, cytosine–phosphate–guanine dinucleotide; cDMG, candidate differentially methylated gene; DEG, differentially expressed gene; DMG, differentially methylated gene; DMR, differentially methylated region; DNA, deoxyribonucleic acid; FDR, false discovery rate; GO, gene ontology; GSEA, gene set enrichment analysis; HC, offspring exposed to maternal obesity (high-fat diet-fed dam); HFD, high-fat diet; KEGG, Kyoto Encyclopedia of Genes and Genomes; logFC, log_2_ fold change; MO, maternal obesity; MOB, blood from maternal obesity offspring; MOK, kidney from maternal obesity offspring; mRNA, messenger ribonucleic acid; NES, normalized enrichment score; ORA, over-representation analysis; PCR, polymerase chain reaction; RRBS, reduced representation bisulfite sequencing; RT-qPCR, reverse transcription quantitative polymerase chain reaction; SEM, standard error of the mean; TSS, transcription start site; WGBS, whole-genome bisulfite sequencing.
