# Supplementary material for: Maternal Obesity Induces DNA Methylation Changes in the Kidneys and Blood of Rodent Offspring at Adulthood
Source: Nutrients. 2026 Sep 29;18(19):3212. doi: 10.3390/nu18193212 (PMC13667289; doi:10.3390/nu18193212)
Supplement: Supplementary file 1 [file nutrients-18-03212-s001.zip › nutrients-4562778-supplementary.pdf]

Supplementary information

Table S1. Mouse-specific primers used in quantitative real-time PCR.

**Gene**	**Forward Primer Sequence**	**Reverse Primer Sequence**
18S	ACCGCAGCTAGGAATAATGGA	GCCTCAGTTCCGAAAACC
Rcsd1	AGGAACTCACCTGCCCTCTTAC	ATGGCTCCACAGCCCTGTAATC
Cdk8	GAGAGTTGTCCCTCCTACCACT	GAGGTAGCTGAGTATCCCATGC
Pex2	TGACAGAACGCCTCCTTGGCAT	CAGAAACTCAGCAAAGCCATGCC
2810004N23Rik	CCAAAGAACGGCAGCAAGCTAG	GGTTAAACTCCTGAATATCCACATC
Rnf150	GCTCCAACACAAACGAGACCATC	CCTTTCCAGCAGGCTCACTATC
Taco1	GAGGCGTTATCAAACAGTGGTCC	CTCTCTGTCCTCCACTCCAACA
Axin2	ATGGAGTCCCTCCTTACCGCAT	GTTCCACAGGCGTCATCTCCTT
1700030C10Rik	CCTGGAGGAATCCGTAAGCACA	TGCCCTCCATCCTGCAAATA
Gm20110	CCTTTTCCTGGACCCTGACT	CCAGCCATCTTCCTGAGGTT
Cdon	TGAAGGACAGCCTGCCATGCTT	CCTGGAGGAATCCGTAAGCACA

Table S2. Genes with differential methylation identified in the kidney.

**Gene**	**Entrez gene ID**	**Chromo-some**	**No. of differentially methylated loci**	**Most significant locus**	**Log FC**	**FDR**
Rnf150	330812	8	1	82819166	2.012587539	1.79E-05
2810004N23Rik	66523	8	1	124861639	1.618804537	0.00064399
Cdon	57810	9	1	35475501	3.759856877	0.04591901
Rcsd1	226594	1	5	165708034	-1.27687654	5.12E-06
Cdk8	264064	5	8	146261015	-0.79472387	1.29E-05
Pex2	19302	3	8	5860683	-0.85407697	2.67E-05
Taco1	70207	11	1	106066796	-0.72064405	0.00038078
Axin2	12006	11	9	109011893	-0.69297453	0.00083811

1700030C10Rik	69513	12	6	20232859	-1.18219665	0.0024305
Gm20110	100504202	10	1	99608836	-2.58152183	0.00807249

Table S3. Genes with differential methylation identified in the blood.

**Gene**	**Gene ID**	**Chromo-some**	**No. of differentially methylated loci**	**Locus**	**Log FC**	**FDR**
Arhgef26	622434	3	2	62159251	3.850341772	1.36E-12
Pcdh7	54216	5	1	58000647	-3.90594127	3.50E-08
SOCS3	12702	11	6	117968116	-2.12267272	0.00184432
Ets1	23871	9	1	32747821	3.4674826	0.00209477
B230209E15Rik	319752	7	1	61569933	-6.34834334	0.00419219
A630010A05Rik	545280	16	1	14502166	-3.96486629	0.00638065
Gm28590	102638832	1	1	122357166	2.769993431	0.02295961
Cylc1	67407	X	1	111043987	1.725073145	0.02519397
H2-Q6	110557	17	1	35425805	1.727383328	0.03418153
Tmtc2	278279	10	1	105238440	2.469182267	0.03733457
Rcsd1	226594	1	5	165708031	-1.98331505	1.03E-05
Cdk8	264064	5	6	146261081	4.956444874	8.38E-33
Pex2	19302	3	1	5860668	-0.6951088	0.03663806
2810004N23Rik	66523	8	1	124861639	2.344801855	1.15E-07

Table S4. Summary of methylation findings and kidney mRNA expression of genes identified to be significantly differentially methylated due to maternal obesity only in the kidney.

**Gene**	**Effect of maternal obesity on gene methylation**	**Effect of maternal obesity on kidney mRNA expression**
Rnf150	increased	no significant change
Taco1	**reduced**	**increased**
Axin2	**reduced**	**increased**
1700030C10Rik	reduced	unchanged

Gm20110	reduced	unchanged
Cdon	**increased**	**Trend to increase**

Table S5. Summary of methylation findings and kidney mRNA expression of genes identified to be significantly differentially methylated in both the kidney and blood due to maternal obesity.

**Gene**	**Methylation**	**mRNA expression**
Rcsd1	**reduced**	**increased**
Cdk8	**reduced in kidney**<br>**increased in blood**	**increased**
Pex2	**reduced**	**trend of increase**
2810004N23Rik	increased	unchanged
